# A Proposed Bill for a State Board of Health*See editorial.

**Published:** 1903-01

**Authors:** 


### TEXAS MEDICAL JOURNAL.

ESTABLISHED JULY, 1885.

PUBLISHED MONTHLY.—SUBSCRIPTION $1.00 A YEAR.

VOL. XVIII. AUSTIN, JANUARY, 1903. No. 7.

### Original Contributions.

### A Proposed Bill for a State Board of Health.*

AN ACT

To carry into effect Section 32 of Article XVI of the Constitution of the State of Texas in relation to a State Board of Health and Vital Statistics; to enlarge the scope and powers of the existing health system of the State for the better protection of the public health; to create and establish a State Board of Health and Vital Statistics in conjunction with the existing Quarantine Department; to create and put in operation County and Municipal Boards of Health; to define the powers, duties and authorities of said boards, and fix the salaries of the officers of the State Board of Health. To authorize the State Board of Health to prepare, promulgate and enforce, under suitable penalties for violations of its provisions, a sanitary code for the State of Texas, and regulations for the record and preservation of its vital statistics; to provide for a general sanitary supervision of counties and municipalities, and to repeal all laws and parts of laws in conflict with this act.

SECTION 1. *Be it enacted by the Legislature of the State of Texas*: That a State Board of Health and Vital Statistics is hereby created and established, to consist of five legally qualified physicians, the Governor and the Attorney General, the State Health Officer under existing laws being ex-officio a member and President of the Board and its executive officer.

*See editorial.

SEC. 2. The Governor shall, by and with the consent of the Senate, on or before the 10th day of May, 1903, appoint the other four members of the Board as follows: (1) A physician learned in sanitation and familiar with statistics, who shall be Registrar of Vital Statistics. He shall, in addition to the duties as a member of the State Board of Health, collect, record, tabulate, and publish in the yearly report of the Board, hereinafter provided for, the vital and mortuary statistics of the State, and shall from time to time, under the direction and supervision of the Board, prepare, publish and disseminate amongst the people for their information and protection, such literature as may enlighten the public upon sanitary matters, and the causes and means of prevention of diseases. During his term of service he shall reside at Austin and give his whole time to the duties of his office. He shall be paid a salary of $2500 per annum. (2) A physician learned in chemistry, pathology and bacteriology, and skilled in the use of the microscope, who shall be the pathologist of the Board. During his term of service he shall reside at Austin and shall give as much of his time as is necessary to the duties of his position; he shall make all examinations and analyses of matters referred or submitted to him by the Board, and shall make his report in writing, to the Board, of all such examinations, which report shall be incorporated in the annual report of the Board hereinafter provided for. He shall receive an annual salary of two thousand dollars. (3) Two physicians of learning and skill, eminent in the profession, from the State at large, who shall be consulting and advisory members of the Board. They shall each be allowed twenty-five dollars per day for each and every day they are in attendance upon meetings of the Board at Austin, including the time spent in transit, and five cents per mile going to and returning from such meetings, to be paid on their voucher, approved by the Governor and the President of the Board, by warrant drawn on the Comptroller against the Fumigating Fund of the Quarantine Department, hereinafter provided for.

SEC. 3. Three members of the Board shall constitute a quorum for the transaction of business. The Board shall meet at Austin on the first Tuesday after appointment and commission, and every three months thereafter on a day to be fixed by the Board. They shall meet on the call of the President or on demand of two members, whenever such meeting is necessary.

The office of the Board shall be in the Capitol at Austin, and the Board shall be furnished with all necessary equipment, books, stationery, blanks, etc., as other departments of the State Government are.

SEC. 4. The President of the Board shall have, as under existing laws, charge of, and superintend the administration of all maritime and international quarantine service, and disinfecting stations for the protection of the State from imported contagious and infectious diseases. He shall perform certain other duties hereinafter stipulated.

SEC. 5. The Board shall be authorized to employ the necessary clerical assistance and to fix and pay the salaries of its employes, as hereinafter provided, including a Secretary, who shall be Secretary for both the Quarantine Department and the State Board of Health.

SEC. 6. The members of the Board of Health shall hold their offices for a term of two years and until their successors are appointed, unless sooner removed for cause. They shall qualify and be commissioned in the manner provided by the laws of Texas for all other officers of the State.

SEC. 7. In addition to the powers and duties prescribed by existing laws for his governance as State Health Officer, the President of the Board shall have power, after the adjournment of the Board and during the time between the meetings of the Board and when the Board is in session, to issue all orders and warrants, and to take all necessary steps to execute the sanitary laws of the State, and rules, ordinances, and regulations of the Board made under authority of this act.

SEC. 8. The Board shall have supervisory power over the care and control of infectious and communicable diseases within the State in order to suppress and prevent the spread of same.

SEC. 9. The Board shall prepare, or cause to be prepared, a sanitary code for the State of Texas, which shall contain and provide rules and regulations and ordinances of a general nature for the improvement and amelioration of the hygienic and sanitary condition of the State. On adoption of said code by the Board it shall be published at length and in full in at least two newspapers in the State, on ten consecutive days, and there shall also be printed and published in pamphlet form such number of copies as may be necessary for distribution for the information of health boards, health and sanitary officers, and the public generally. When so printed said code shall cover and provide for, especially, land and maritime quarantine regulations; the reporting, care and management of infectious, contagious and communicable diseases; it shall regulate the manner of keeping and reporting and tabulating vital and mortuary statistics; it shall provide for affording facilities for vaccination, provided the same shall not be made compulsory except in cases of children attending public schools; it shall regulate the carriage and transportation of persons, freight and dead bodies brought into the State or transported through or into the State so far as the same may affect the public health; it shall provide for the carrying out of the laws of the State in regard to the adulteration of articles intended for human food or consumption; it shall provide for the inspection of meats, milk, coal oil and other articles affecting the public health and safety where and when the same may be brought from one county into another, or from outside of the State, leaving to local boards hereinafter provided, the regulation of the sale or offering for sale of said articles within the county or municipality to which the same may be brought, and said code shall contain general rules in regard to such health, sanitary and hygienic subjects, as can not, in the opinion of the State Board of Health, be efficiently and effectively regulated by the local boards. It shall devise and execute under suitable penalties for violation thereof, ordinances to prevent the pollution of all water supplies and streams. It shall make and have power to enforce, under penalties fixed by the Board, ordinances to regulate sanitary policing of premises public or private, jails, hospitals, public buildings, shops, factories, slaughter houses and yards, for the removal of possible causes of disease; to abate nuisances; to cause disinfection of all infected houses, public or private, and of sleeping cars and other railroad cars, and of all public conveyances, these means of prevention to be taken by the owners of said premises or conveyances at their own cost.

SEC. 10. The Board shall have power to appoint inspectors when necessary and fix the salaries thereof. All inspectors and officers of the said Board shall have power to arrest without warrant all persons violating the provision of any rule or regulation of the said Board, when such violation has occurred or is occurring within the sight, view or personal knowledge of such inspector or officer; and in all cases where such violation may not have occurred within the sight, view or personal knowledge of said inspector or officer, said functionary shall have only the right to arrest in execution of a warrant duly issued by the President of the Board.

It is hereby made the duty of all sheriffs, their deputies, constables and their deputies, police officers of towns and cities, and all other peace officers, to assist in the arrest or apprehension of all persons violating the articles of any rule or regulation of the State Board of Health; to themselves arrest and apprehend all offenders committing the offenses in their view or sight, or within their personal knowledge.

SEC. 11. The members of the State Board of Health and every person duly authorized by them may, without fear or hindrance, enter, examine and inspect all grounds, erections, vehicles, structures, public buildings and places.

SEC. 12. Be it further enacted that each and every organized county in the State shall create and establish a board of health in the following manner: The County Commissioners Court of each county in the State shall, immediately after the promulgation of this act, appoint a resident duly licensed physician of the county, of recognized character and standing, to be County Health Officer, provided there be at the time no such officer as provided for under the quarantine law; and in default of making such appointment within thirty days after notice to do so by the State Board of Health the Board shall appoint a County Health Officer.

The county physician shall be president of the County Board of Health, which shall be composed of the Clerk of the County Court and the County Judge and the County Health Officer. They shall serve two years from date of appointment and shall be paid by the county such salary as may be agreed on.

Said Board shall have sanitary supervision of all the territory embraced in their county outside the limits of incorporated towns and cities, which shall be under the jurisdiction of their respective municipal boards hereinafter provided for. Every County Board of Health shall meet on the first Tuesday after the appointment of its members, and monthly thereafter or as often as necessary. The County Boards of Health shall exercise the powers and perform the duties prescribed for them by the Commissioners Court, and shall also enforce the rules and regulations prescribed by the State Board of Health for the protection of the public health, the prevention and suppression of diseases, and shall be subject to the State Board of Health and obey its instructions, in default of which they shall be fined in a reasonable sum, at the discretion of the State Board for neglect or failure to do so, and the county health officer may be removed by said State Board, for continued or repeated neglect of duty or incompetency. In event of such removal the State Board of Health shall appoint the county health officer from amongst the licensed physicians of the county.

SEC. 13. Be it further enacted, etc., that the council or legislative body of each and every incorporated municipal government in the State shall establish and organize a town or city board of health in the manner following:

The said council or legislative body shall, immediately after the promulgation of this act, or as soon thereafter as may be practicable, elect or apoint three persons of said municipality to be members of the municipal board of health. Such persons shall not be members of the said council or occupy any other office in the said municipality, and two of the persons so elected shall, if practicable, be duly registered and licensed physicians. Said persons so elected or appointed shall constitute the city or town board of health, and shall serve for two years from the date of their qualification. For the cities of Galveston and Houston the Governor shall appoint, with the advice and consent of the Senate, one member on each of said city boards. The council or legislative body shall provide ample means for the maintenance and operation of said board. Said town or city boards of health shall meet on the first Tuesday after the commission of its members and shall elect a chairman, who shall be a duly registered and licensed physician, who shall be health officer of the town or municipality, who shall serve in said capacity, exercise the powers and perform the duties usual and incident to such officers in similar organizations. The chairman shall receive such an annual salary as the council of said municipality may fix and pay. The said board of health shall have power to appoint a sanitary officer, whose duties shall be to enforce the requirements of said Board in all matters of sanitation, and also to act as Secretary. His salary shall be fixed and paid by the Board.

SEC. 14. Be it further enacted, etc., that all members of county and municipal boards shall have been at the time of their appointment or election, two years residents and voters of the county or municipality in which they are to act as said boards, and the membership shall be vacated on the removal therefrom of the member. No member of said board, with the exception of the chairman and sanitary officer, shall receive any pay or emolument in any way for the services rendered as a member of the Board, and no member shall be in any way directly or indirectly interested in any contract for supplies to be furnished or services to be rendered to said Board.

SEC. 15. Be it further enacted, etc., that said county and municipal boards of health shall have power and authority to pass health and sanitary ordinances for defining and abating nuisances dangerous to the public health; to regulate drainage and ventilation, with reference to human habitations and places of business and public resort; to regulate the carrying on of trade and business injurious to public health; for the disposition of fecal matter and garbage; to regulate the erection of buildings, with due regard to the filling of lots and the grading thereof, and the arrangement of said building; for the vacation of, disinfection of, or demolition of buildings, when necessary for the protection of public health; for the registration of births, deaths and marriages, and the keeping of vital statistics to be registered and reported to the State Board of Health, under its instructions and regulations, and generally all health and sanitary ordinances necessary and incident to proper local sanitation of the county, city or town in which they exercise their powers.

They shall act under the supervision and advice of the State Board of Health, and shall pass no ordinance in conflict or inconsistent with the powers and duties of the State Board of Health, and furnish such other information as the State Board of Health may require, the same to be embodied in the annual report of the State Board. Local boards shall have power to establish quarantines with the co-operation of the councils of municipalities and the commissioners court. The State Board shall have supervisory power over all local quarantines so established.

SEC. 16. All necessary expenses, costs and charges of local sanitation shall be borne by the county, city or town in which the local board shall be established, and in case the fiscal authorities shall refuse to budget for, appropriate or pay the same, the local boards shall have the right to the writ of mandamus before a court of competent jurisdiction to compel the proper action by said county, city or town authority.

SEC. 17. Be it further enacted, etc., that in the event that any case shall be reported to, or come to the knowledge of any local board, which is either deemed to be a case of contagious or infectious disease, or suspected of so being, the local board shall immediately isolate the same and communicate the fact by the most expeditious means at hand to the State Board of Health, together with the information as to what steps have been taken by the local board to isolate and care for the same, and shall from time to time communicate the progress of the case and disease to the State Board of Health. On receipt of such information by the said Board of Health, the President of said Board shall, if he deem the emergency sufficient and necessary, send an expert physician to be selected by him, to examine and diagnose the disease, and if on such examination and diagnosis the expert shall declare the case to be one of obnoxious and infectious nature, liable to spread or to become dangerous to the general public health of the State, the State Board of Health or its President shall instruct the health officer of the board of health of the county, city or town, as to what steps to take, if any shall be taken, to isolate the case and prevent the spread of the contagion or infection therefrom and shall require that the local health officer shall immediately conform thereto and put the same in operation. In the event that said local authorities shall fail or neglect to so act within a reasonable time, or in so acting shall fail to do so in a manner satisfactory to the State Board, the Board, or its President, shall take charge of the case and manage the same through its own officers and employes.

All expenses, costs and charges incurred in the management, control and supervision of such cases shall be borne and paid by the county, town or city in which the case may be, and the failure of the county or municipality to reimburse the State Board for the amount of its expenditures incurred in such cases, the State Board of Health shall have the right to proceed by writ of mandamus in any court of competent jurisdiction to compel the payment of the same.

In case that the county, town or city, or any portion thereof, shall become infected with any contagious or infectious disease, to such an extent as to threaten the spread of such disease to other portions of the State, the State Board of Health shall issue its proclamation declaring the facts and ordering it in quarantine, and shall order the local boards of health in other counties, towns and cities, to quarantine against said locality and shall establish and promulgate the rules and regulations, terms and conditions on which intercourse with said infected locality shall be permitted, and shall issue to the other local sanitary authorities instructions as to the measures adopted in quarantining against persons, goods, or other property coming from said infected localities and these rules and regulations shall be observed and obeyed by all other health authorities, provided that should any other non-infected portion of the State desire to add to the regulations and rules, terms and conditions already imposed by the State Board, they may do so, on approval of the State Board of Health.

The State Board of Health may, in its discretion, prohibit the introduction into any infected portion of the State, persons acclimated, or unacclimated, or said to be immune, when in its judgment the introduction of such persons would add to or increase the prevalence of the disease. The State Board of Health shall render to the local boards of health all the assistance in their power and which the conditions of their finances will permit.

SEC. 17. All physicians, surgeons or accouchers who may attend at the birth of a child; or in the absence of such attendance, any other party having knowledge of the same, shall report the fact to the clerk of the county court, together with the race to which it belongs, and whether legitimate or otherwise, foreign or native parents, still born or alive, within ten days after said birth occurs, under a penalty of five dollars for each failure to do so, to be collected as other fines for misdemeanors are.

All physicians, surgeons, accouchers, coroners, or in the absence of these, any person who shall become cognizant of a death, shall report the same together with the race, nativity, sex, age, residence, whether alien or citizen, and the cause of death, to the clerk of the county court within ten days after the occurrence, under a penalty of not less than five dollars nor more than fifty dollars for each failure to do so, these data to be recorded as a part of the vital statistics of the county and State. Said county or city health officers of the county or city boards of health shall superintend the collection of the vital statistics of the county, and the clerk of the county court shall keep a record of the same in connection with and supplemental to the records of marriages now provided by law.

All expenses of the State Board of Health, including the salary and pay and per diem of its members, shall be paid out of the Fumigating Fund of the Quarantine Department on a proper voucher approved by the Governor and the President of the Board, as other payments are made.

Sec. 18. Be it further enacted, etc., that all laws and parts of laws in conflict with, inconsistent with, or superceded by the provisions of this act are hereby repealed, but all laws or parts of laws, or city ordinances, State Board of Health ordinances, State and municipal rules and regulations now existing and not in conflict with, or inconsistent with or superceded by the provisions of this act are continued in full force and effect, and this act shall not be construed or interpreted so as to deprive the State health officer, or the local boards of health, of any powers or authorities they may have under existing laws, except in so far as these powers and authorities may be modified or changed by the provisions of this act, and then only to the extent of the modification or change.

Sec. 19 The Governor may remove any member of the State Board of Health for cause.

Sec. 20. The State Board of Health shall make and publish a full report of its work on the first of September of each year, as all other reports are made to the Governor and the Legislature.

The fact that there is no uniform and efficient law for the suppression of diseases other than those of foreign origin and no system of preserving the vital statistics of the State creates an emergency and an imperative public necessity that the constitutional rules requiring bills to be read on three several days be suspended, and that this act take effect and be in force from and after its passage, and it is so enacted.

